# *LDB1* overexpression is a negative prognostic factor in colorectal cancer

**DOI:** 10.18632/oncotarget.12481

**Published:** 2016-10-05

**Authors:** Sebastián A. García, Anka Swiersy, Praveen Radhakrishnan, Vittorio Branchi, Lahiri Kanth Nanduri, Balázs Győrffy, Alexander M. Betzler, Ulrich Bork, Christoph Kahlert, Christoph Reißfelder, Nuh N. Rahbari, Jürgen Weitz, Sebastian Schölch

**Affiliations:** ^1^ Department of Gastrointestinal, Thoracic and Vascular Surgery, Medizinische Fakultät Carl Gustav Carus, Technische Universität Dresden, Fetscherstr. 74, 01307 Dresden, Germany; ^2^ Department of General, Gastrointestinal and Transplantation Surgery, University Hospital Heidelberg, Ruprecht-Karls-Universität Heidelberg, Im Neuenheimer Feld 110, 69120 Heidelberg, Germany; ^3^ Department of General, Gastrointestinal, Thoracic and Vascular Surgery, University Hospital Bonn, 53127 Bonn, Germany; ^4^ MTA TTK Lendület Cancer Biomarker Research Group, Magyar Tudósok körútja 2., H-1117, Budapest, Hungary; ^5^ Semmelweis University, 2nd Department of Pediatrics, Bókay u. 53-54., H-1083, Budapest, Hungary

**Keywords:** ldb1, wnt signaling, colorectal cancer, proximal, distal

## Abstract

**Background:**

Colorectal cancer (CRC) is the third most common cancer in western countries and is driven by the Wnt signaling pathway. LIM-domain-binding protein 1 (*LDB1*) interacts with the Wnt signaling pathway and has been connected to malignant diseases. We therefore aimed to evaluate the role of *LDB1* in CRC.

**Results:**

Overexpression of *LDB1* in CRC is associated with strikingly reduced overall and metastasis free survival in all three independent patient cohorts. The expression of *LDB1* positively correlates with genes involved in the Wnt signaling pathway (*CTNNB1*, *AXIN2*, *MYC* and *CCND1*). Overexpression of *LDB1* in CRC cell lines induced Wnt pathway upregulation as well as increased invasivity and proliferation. Upon separate analysis, the role of *LDB1* proved to be more prominent in proximal CRC, whereas distal CRC seems to be less influenced by *LDB1*.

**Materials and Methods:**

The expression of *LDB1* was measured via RT-qPCR in 59 clinical tumor and normal mucosa samples and correlated to clinical end-points. The role of *LDB1* was examined in two additional large patient cohorts from publicly available microarray and RNAseq datasets. Functional characterization was done by lentiviral overexpression of *LDB1* in CRC cell lines and TOP/FOP, proliferation and scratch assays.

**Conclusions:**

*LDB1* has a strong role in CRC progression, confirmed in three large, independent patient cohorts. The *in vitro* data confirm an influence of *LDB1* on the Wnt signaling pathway and tumor cell proliferation. *LDB1* seems to have a more prominent role in proximal CRC, which confirms the different biology of proximal and distal CRC.

## INTRODUCTION

Colorectal cancer (CRC) is one of the most common cancer entities worldwide [1]. Survival rates are highly dependent on the occurrence of distant metastases [2]. Several signal pathways are known play a role in CRC development and metastasis, most prominently the Wnt signaling pathway, which is altered in 93% of all CRC tumors [3]. During the activation of the Wnt pathway, Wnt ligands bind to Frizzled receptors to induce the phosphorylation of LRP coreceptors [4]. A complex formed by Axin, APC, CK1α (serine/threonine kinases casein kinase 1 alpha) and GSK3 (glycogen synthase kinase 3) is recruited to the membrane and hampers the ubiquitination of phosphorylated β-Catenin (*CTNNB1*), preventing its degradation [5]. β-Catenin thus accumulates in the cytosol and translocates into the nucleus, where it induces the transcription of *MYC*, *CCND1*, *CD44* and *AXIN2* genes [6–9].

LIM domain binding protein 1 (LDB1, also known as CLIM2 and NLI) is an ubiquitous nuclear adaptor protein working as a transcriptional modulator [10]. In mice, intrauterine knockout of *Ldb1* causes severe anterior-posterior patterning defects, including anterior truncation and posterior duplication, which can be partially explained by a downregulation of Wnt pathway antagonists [11–13]. In adult mice, tamoxifen-induced knockout of *Ldb1* results in drastic changes in the small intestine including a loss of Lgr5^+^ intestinal stem cells and significant activation of the Wnt pathway [14]. In a chemically induced mouse model of hepatocellular carcinoma, hepatocyte-directed *Ldb1* knockout lead to larger and more frequent tumors, which also displayed increased proliferation and resistance to apoptosis. Microarray and RT-PCRs assays from tumor cDNA confirmed Wnt activation in *Ldb1*-deficient tumors [15]. Taken together, the currently available data indicates a prominent role of *Ldb1* in Wnt signaling and murine intestinal homeostasis as well as inhibitory effects of *Ldb1* on (hepatocellular) tumorigenesis.

Despite the role of *LDB1* in intestinal homeostasis and Wnt signaling, there is no data available on the role of *LDB1* in CRC. We therefore aimed to evaluate the role of *LDB1* in human CRC and investigate its molecular effects on colorectal tumorigenesis. To this end, we evaluated the expression of *LDB1* in CRC patient samples and correlated the *LDB1* expression to clinical parameters. Moreover, functional assays in cell lines overexpressing *LDB1* were used to investigate the molecular effects of *LDB1* in CRC.

## RESULTS

### High *LDB1* expression in CRC is associated with decreased overall and metastasis-free survival

In an initial screening attempt to identify a possible role of *LDB1* in CRC, *LDB1* transcripts were measured in primary tumor and normal mucosa samples from 59 CRC patients of all stages (Table 1), who underwent colorectal tumor resection at the Department of Surgery of University Hospital Heidelberg. Tumor Ct values were normalized to the corresponding mucosa and patients were separated into two groups according to a higher or lower expression of *LDB1* in comparison with the mucosa. As cutoff for high *LDB1* expression ΔΔCt > 0.5 was chosen, resulting in two cohorts of 46 and 13 patients, respectively. Patients were followed for a median of 30 (0–74) months.

**Table 1:** Patient characteristics

Clinicopathologic characteristics		Total		*LDB1_low_*		*LDB1_high_*	
		N°	%	N°	%	N°	%
**Patients**		59	100.0	46	69.5	13	30.5
**Age**		63.8		63.5		64.8	
**Gender**	Male	29	49.2	23	79.3	6	20.7
	Female	30	50.8	23	76.7	7	23.3
**Localization**	Colon	24	40.7	17	70.8	7	29.2
	Rectum	35	59.3	29	82.9	6	17.1
**UICC stage**	I	10	16.9	10	100.0	0	0.0
	II	18	30.5	13	72.2	5	27.8
	III	19	32.2	15	78.9	4	21.1
	IV	12	20.3	8	66.7	4	33.3
**Grade**	−	4	6.8	3	75.0	1	25.0
	2	45	76.3	36	80.0	9	20.0
	3	10	16.9	7	70.0	3	30.0

**Figure 1:**
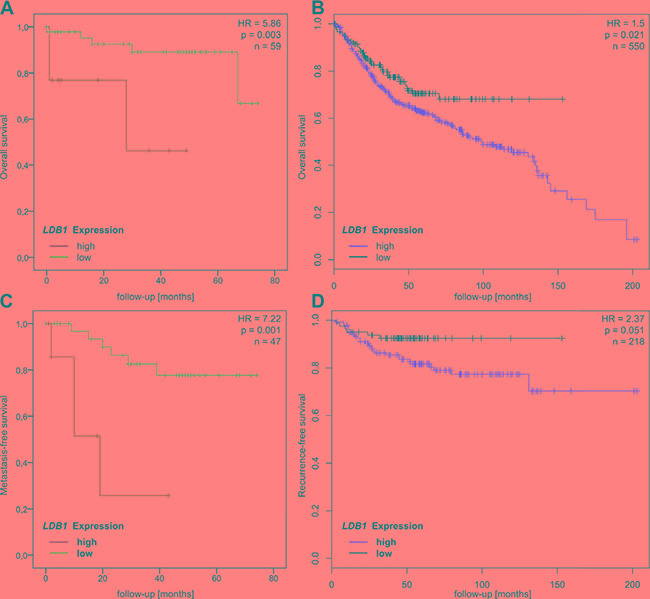
(**A** and **B**) Overall survival of CRC patients with high and low *LDB1* expression in the Heidelberg cohort (A, HR = 5.86, *p* **=** 0.003) and the Affymetrix cohort (B, HR = 1.5, *p* = 0.021). (**C** and **D)** Recurrence-free survival of CRC patients with high and low *LDB1* expression in the Heidelberg cohort (C, HR = 7.22, *p* = 0.001) and the Affymetrix cohort (D, HR = 2.37, *p* = 0.051).

In contrast to previous data [14, 15], high *LDB1* expression in the primary tumor was significantly associated with decreased overall survival (66.2 vs. 31.5 months, Hazard Ratio (HR) = 5.86, *p* = 0.003) (Figure 1A) in our cohort of CRC patients. In order to validate these results, we analyzed the influence of *LDB1* expression (measured via Affymetrix microarray) on overall survival in a publically available, independent cohort of 550 CRC patients of all stages. Overall survival was again shorter in tumors overexpressing *LDB1* (HR = 1.5 (1.1–2.1), *p* = 0.021, Figure 1B). Further validation could be achieved by exploring the role of *LDB1* overexpression in the TCGA cohort [3], in which *LDB1* expression was measured via RNA sequencing. In this cohort (*n* = 267), *LDB1* overexpression again lead to a significantly reduced overall survival in CRC patients of all stages (HR 2.1 (1.0 – 4.1), *p* = 0.038, Supplementary Figure S1A).

To evaluate the influence of *LDB1* expression on systemic tumor dissemination, we performed a subgroup analysis on patients with non-metastatic disease only. This analysis revealed a significantly reduced metastasis-free survival (62.8 vs. 19.7 months, HR = 7.22, *p* = 0.001, Figure 1C) in patients with high *LDB1* expression in the primary tumor. In the Affymetrix cohort of non-metastatic patients (*n* = 218), recurrence-free survival was also reduced in patients with *LDB1*-overexpression tumors (HR = 2.37 (0.97–5.78), *p* = 0.051, Figure 1D). The TCGA clinical data does not include recurrence-free survival on non-metastatic patients; we therefore analyzed only overall survival in this cohort (*n* = 176), which again showed unfavorable prognosis in *LDB1*-overexpressing tumors (HR 4.0 (1.1–13.7), *p* = 0.019, Supplementary Figure S1B).

In conclusion, *LDB1* overexpression proved to be an unfavorable prognostic factor in three large, independent patient cohorts. *LDB1* overexpression also seems to be strongly associated with early recurrence in non-metastatic patients.

### Tumor *LDB1* expression positively correlates with Wnt pathway activation in CRC patient samples

As *LDB1* surprisingly turned out to be a negative prognostic factor in CRC and as *LDB1* has previously been associated with the Wnt signaling pathway [13–15], we next aimed to investigate its influence on the Wnt signaling pathway in CRC.

**Figure 2:**
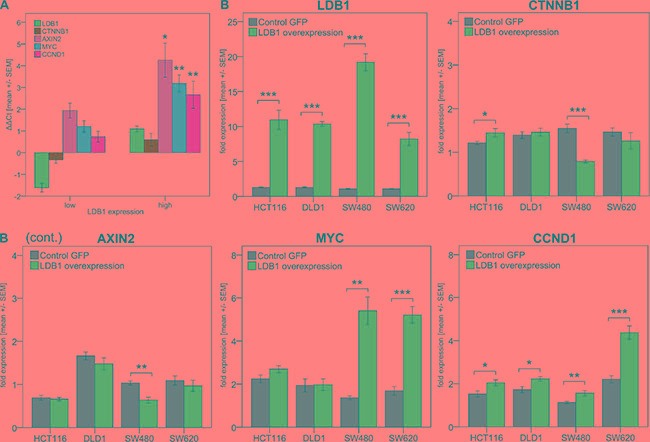
(A) *LDB1* and Wnt pathway genes expression in CRC-patients with high vs. low *LDB1* expression levels. *LDB1*_high_ patients showed higher expression of Wnt pathway genes. (**B**) Expression of *LDB1* and Wnt pathway genes after lentiviral *LDB1* upregulation in CRC cell lines. Values were calculated using the 2^ΔΔCt^ formula. Displayed are means +/− SEM. (*) *p* < 0.05, (**) *p* < 0.01, (***) *p* < 0.001.

To this end, the expression of *LDB1* was correlated with the expression of the Wnt signaling-associated genes *CTNNB1*, *AXIN2*, *MYC* and *CCND1* in patient primary tumor samples (Figure 2A). Upon direct comparison in patients with high or low *LDB1* expression, *CTNNB1* showed a strong tendency towards overexpression (*p* = 0.11), wheres *AXIN2*, *MYC* and *CCND1* were significantly overexpressed (*p* < 0.05 for all three genes) in *LDB1*-overexpressing tumors. In addition, we observed a highly significant positive correlation (*p* < 0.001 for all four genes) between the tumor *LDB1* expression and the expression of Wnt signaling-associated genes (Table 2). This indicates a coincidence of Wnt activation and *LDB1* overexpression, thus explaining the negative prognostic value of *LDB1* overexpression demonstrated in the patient cohorts.

**Table 2:** Correlation between tumor *LDB1* expression and Wnt signaling-associated genes expression in CRC patients

Genes	Pearson correlation	*p* value
***CTNNB1***	0.471	< 0.001
***AXIN2***	0.465	< 0.001
***MYC***	0.448	< 0.001
***CCND1***	0.446	< 0.001

### Upregulation of *LDB1* induces changes in *MYC* and *CCND1* expression

**Figure 3:**
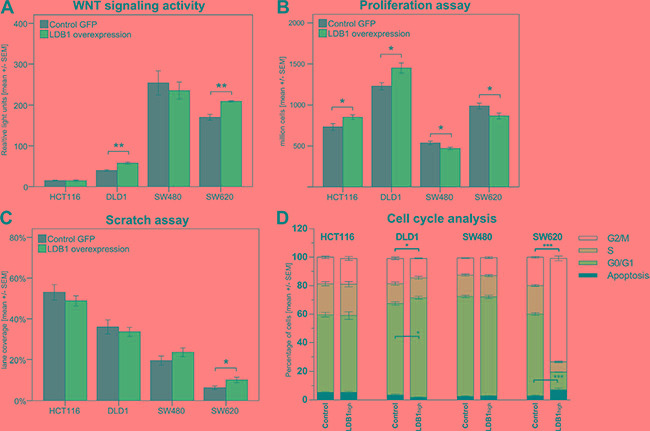
(A) Wnt signaling activity in cell lines overexpressing *LDB1* vs. wild-type cell lines measured with TOP/FOP reporters. Data is shown as luminescence ratio in relative light units. (**B**). Proliferation of CRC cell lines after upregulation of *LDB1*. (**C**) Scratch assay of CRC cell lines. Results displayed as percentage of the scratch covered after 24 hours. (**D**). Cell cycle analysis of CRC cell lines overexpressing *LDB1*. Displayed are means +/− SEM. (*) *p* < 0.05, (**) *p* < 0.01, (***) *p* < 0.001.

As the positive correlation between *LDB1* expression and Wnt signaling in tumor samples was at least partly contradictory to previously published data [13–15], we aimed to investigate this interaction in detail. To this end, 4 human CRC cell lines (HCT116, DLD1, SW480, SW620) were transduced with a third generation lentiviral vector to generate cell lines stably overexpressing *LDB1* (Figure 2B, Figure 3A–3D). The expression of *LDB1* was incremented 7 to 18 fold in comparison to wild-type cells. The 4 cell lines were chosen as they represent different stages, grading and sites of origin of CRC manifestations.

The influence of this overexpression on Wnt signaling (namely the expression of Wnt signaling-associated genes *CTNNB1*, *AXIN2*, *MYC* and *CCND1)* was measured via qPCR (Figure 2B). The general trend pointed towards an upregulation of Wnt signaling in the *LDB1*-overexpressing cell lines; however, we also observed differential effects of *LDB1* on the expression of Wnt signaling-associated genes. All cell lines overexpressing *LDB1* significantly upregulated *CCND1* transcripts; SW620_*LDB1-High*_ cells showed the highest increase with 1.98 fold (2.21 ± 0.46 (SW620_wild-type_) vs. 4.37 ± 0.76 (SW620_*LDB1-High*_), *p* < 0.001). In SW480_*LDB1-High*_ and SW620_*LDB1-High*_ cells, *MYC* displayed an upregulation of 3.96 fold (1.37 ± 0.21 (SW480_wild-type_) vs. 5.40 ± 1.69 (SW480_*LDB1-High*_), *p* = 0.001) and of 3.09 fold (1.69 ± 0.57 (SW620_wild-type_) vs. 5.21 ± 0.95 (SW620_*LDB1-High*_), *p* < 0.001) respectively. The expression of *MYC* in HCT116_*LDB1-High*_ and DLD1_LDB1-High_ cells was not significantly modified. Additionally, SW480_*LDB1-High*_ cells produced a 1.96 fold downregulation of *CTNNB1* transcripts (1.55 ± 0.24 (SW480_wild-type_) vs. 0.79 ± 0.09 (SW480_*LDB1-high*_), *p* < 0.001) and a 1.62 fold reduction of *AXIN2* (1.03 ± 0.12 (SW480_wild-type_) vs. 0.64 ± 0.18 (SW480_*LDB1-high*_), *p* = 0.001) (Figure 2B).

### Effects of *LDB1* overexpression on Wnt signaling activity

To directly examine the stimulation of Wnt signaling activity by *LDB1* in CRC, the previously chosen 4 CRC cell lines were transfected with a TCF-firefly reporter construct indicating Wnt signaling activity via bioluminescence activity. Renilla-luciferase plasmids were used to estimate cell viability [24]. Results are displayed in relative light units of luminescence (Figure 3A). DLD1_*LDB1-High*_ and SW620_*LDB1-High*_ cells exhibited a significant increase of Wnt signaling activity demonstrated by a change from 39.77 ± 4.34 to 57.57 ± 5.81 (*p* = 0.003) and 170.11 ± 12.15 to 209.44 ± 2.66 (*p* = 0.005) relative light units respectively. The Wnt signaling activity did not significantly change in HCT116_*LDB1-High*_ and SW480_*LDB1-High*_ cells.

### Effects of *LDB1* overexpression on proliferation, migratory potential and cell cycle

Cell proliferation was studied by counting the number of cells generated after an incubation time of 72 hours (Figure 3B). The proliferation of HCT116_*LDB1-High*_ and DLD1_*LDB1-High*_ cells was significantly increased compared to their wild-type counterparts: Upon *LDB1* overexpression, HCT116 proliferation increased to 116% (7.33 x 10^5^ ± 1.03 x 10^5^ (HCT116_wild-type_) vs. 8.52 x 10^5^ ± 0.72 x 10^5^ (HCT116_*LDB1-High*_), *p* = 0.042). DLD1 proliferation increased to 118% (1.23 × 10^6^ ± 0.11 × 10^6^ (DLD1_wild-type_) vs. 1.45 × 10^6^ ± 0.15 × 10^6^ (DLD1_*LDB1-High*_), *p* = 0.017). Surprisingly, in SW480_*LDB1-High*_ and SW620_*LDB1-High*_ cells, the proliferation rate was significantly decreased. In both SW480 and SW620 *LDB1* overexpression resulted in a reduction in proliferation by 12% (5.37 x 10^5^ ± 0.55 x 10^5^ (SW480_wild-type_) vs. 4.71 × 10^5^ ± 0.36 x 10^5^ (SW480_*LDB1-High*_), *p* = 0.035; 9.89 × 10^5^ ± 0.86 x 10^5^ (SW620_wild-type_) vs. 8.68 × 10^5^ ± 0.87 × 10^5^ (SW620_*LDB1-High*_), *p* = 0.036).

As we observed increased metastatic activity of *LDB1*-overexpressing tumors, we next aimed to assess the invasiveness of cells overexpressing *LDB1* in a wound healing assay. A scratch was made on the surface of 6-well plates and the width of this lane was measured immediately and after 24 hours. Results are expressed in percentage of the lane covered by cells (Figure 3C). HCT116_*LDB1-High*_ and DLD1_*LDB1-High*_ did not show significant changes in their invasivity (53.04% ± 9.24% (HCT116_wild-type_) vs. 48.86% ± 6.02% (HCT116_*LDB1-High*_), *p* = 0.375; 36.00% ± 8.57% (DLD1_wild-type_) vs. 33.63% ± 5.33% (DLD1_*LDB1-High*_), *p* = 0.578). In SW480_*LDB1-High*_ the covered space was slightly increased (19.53% ± 5.30% (SW480_wild-type_) vs. 23.59% ± 5.38% (SW480_*LDB1-High*_), *p* = 0.218); in SW620_*LDB1-High*_ the invasivity was significantly increased (6.29% ± 2.13% (SW620_wild-type_) vs. 10.16% ± 3.30% (SW620_*LDB1-High*_), *p* = 0.036).

To investigate the effects of *LDB1* overexpression on cell cycle regulation, the cell cycle phases of CRC cell lines were analyzed via flow cytometry (Figure 3C). HCT116_*LDB1-High*_ and SW480_*LDB1-High*_ cells did not show major differences in comparison with wild-type cells. DLD1_*LDB1-High*_ cells displayed a significant increase of cells in G0/G1 phase from 64.04% to 69.72% (*p* = 0.025), indicating an increased number of cells entering proliferation and thus confirming the results of the proliferation assays. This change simultaneously reduced the number of cells in G2/M from 17.73% to 13.64% (*p* = 0.010). Apoptotic cells decreased from 3.43% to 1.83% (*p* = 0.144) and cells in S phase remained constant at 14% (*p* = 0.992). In SW620_*LDB1-High*_ cells, apoptosis augmented from 2.82% to 7.14% (*p* = 0.029), the G0/G1 phase was reduced from 57.35% to 12.45% (*p* < 0.001) and the S phase changed from 20.05% to 7.77% (*p* < 0.001). The G2/M phase increased from 19.78% to 72.64% (*p* < 0.001), indicating an arrest of these cells before mitosis. This observation again confirms the results of the proliferation assays.

### *LDB1* may have different roles in tumors of the proximal and distal colorectum

During embryologic development, the colorectum is formed by two primordial organs, the midgut and the hindgut. The point of fusion is the left colic flexure (Cannon-Böhm's point), where the blood supply switches from the superior mesenteric artery to the inferior mesenteric artery. Multiple studies are currently investigating molecular differences between tumors of the proximal colon (proximal to the left colic flexure) and distal colon (distal to the left colic flexure down to the anal verge). Little is known so far; however, the currently available data indicates that proximal and distal tumors are in fact entirely different entities [28–30]. Interestingly, recent data suggests that the proximal colon is more susceptible to Wnt associated tumorigenesis, which results in tumor formation upon moderate Wnt activation. This increased susceptibility distinguishes the proximal from the distal colon, which requires more pronounced Wnt activation in order to result in tumor formation [31, 32]. As we have demonstrated the role of *LDB1* in Wnt signaling and have also observed differential effects of *LDB1* on CRC proliferation and invasiveness, we hypothesized that the role of *LDB1* may differ between tumors of different locations within the colorectum.

**Figure 4:**
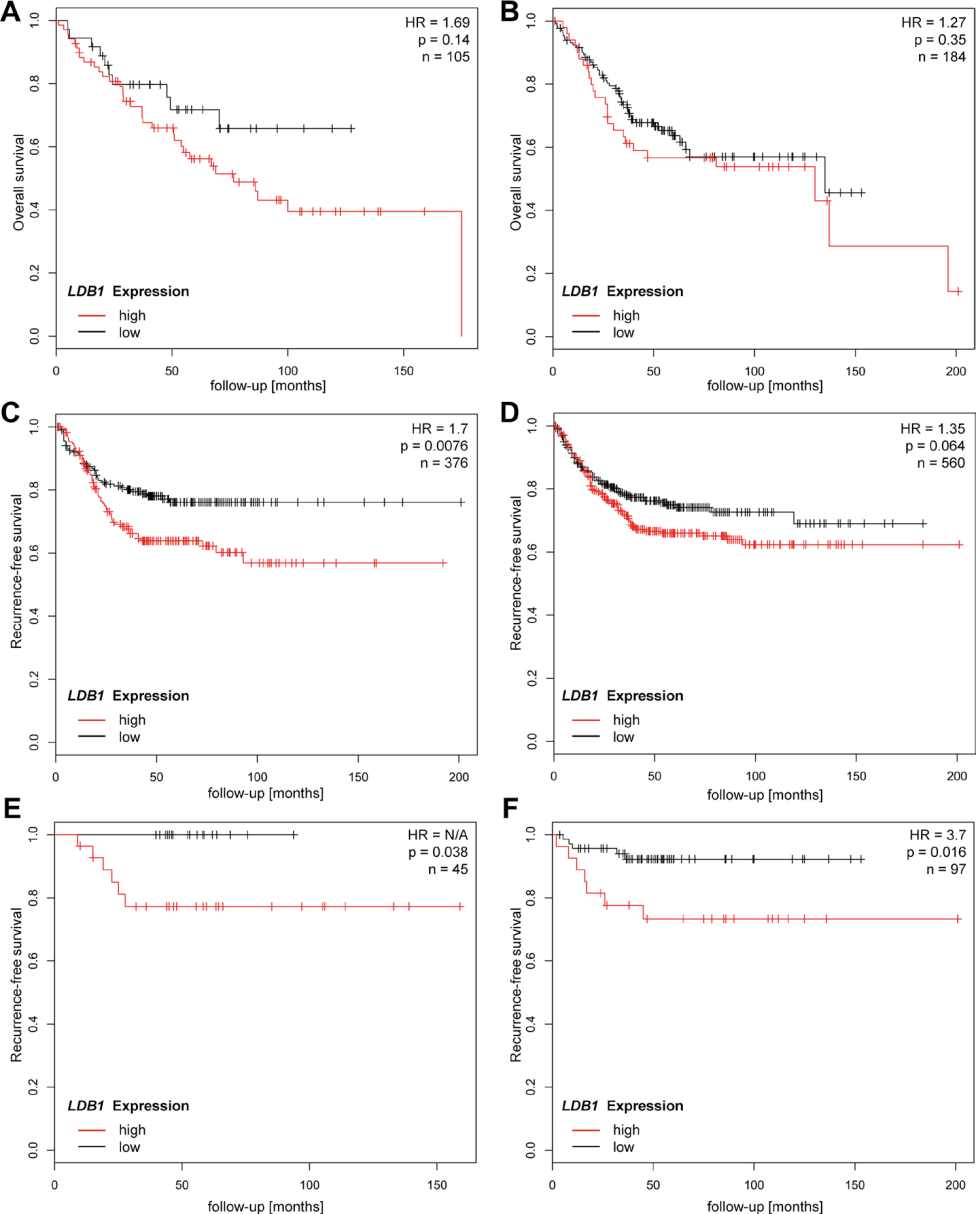
Comparison of survival and recurrence rates depending on tumor location proximal (graphs on left side) and distal to the left colic flexure (graphs on right side) CRC patients of all stages with tumors located in the *proximal* colon and high expression of *LDB1* showed a tendency to shorter overall survival (**A**, HR 1.69, *p* = 0.14) and significantly shorter recurrence-free survival (**C**, HR = 1.7, *p* = 0.0076). *LDB1*_low_Patients with non-metastatic (M0) CRC in the proximal colon showed a strikingly improved recurrence free survival with no recorded recurrences in the study cohort (**E**, HR not calculable, *p* = 0.038). The effects of *LDB1* seem to be much less pronounced in the distal colon; overall survival rates of distal CRC patients are similar for *LDB1*_high_ and *LDB1*_low_ patients (**B**, HR = 1.27, *p* = 0.35). The effects of *LDB1* expression on recurrence-free survival are also less pronounced in patients with distal CRC (**D**, HR = 1.35, *p* = 0.064). In patients with non-metastatic distal CRC, *LDB1* overexpression leads to reduced recurrence-free survival, albeit to a lesser extent than in the proximal colon (**F**, HR = 3.7, *p* = 0.016).

We therefore analyzed the Affymetrix cohort according to tumor localization (Figure 4). The effects of *LDB1* were more pronounced in the subgroup with tumors in the proximal tumor than in the subgroup with distal CRC (OS: HR = 1.69, *p* = 0.14 (proximal) vs. HR = 1.27, *p* = 0.35 (distal); RFS: HR = 1.7, *p* = 0.0076 (proximal) vs. HR = 1.35, *p* = 0.064 (distal)). Stratified analyses based on tumor location were not possible in the Heidelberg patient cohort due to low case numbers. We also evaluate the influence of *LDB1* on recurrence-free survival in non-metastatic patients according to tumor localization and again observed a striking effect of *LDB1* in the proximal colon; in this cohort (*n* = 45) no recurrences were observed in patients with low *LDB1* expression (Figure 4E and 4F).

After splitting the patient samples due to localization, the association between tumor *LDB1* expression and Wnt pathway gene expression remained significant for almost all genes analyzed in samples from the proximal colon and for all genes in samples derived from the distal colon (Table 3A). In samples from the proximal colon, the association between *LDB1* expression on Wnt signaling-associated genes seemed generally to be even stronger than in distal tumors; however, due to the relatively low case number (due to the lower incidence of proximal CRC) significance levels were lower. We also hypothesized that as the rectum is the most distal segment of the colon, the effects of *LDB1* in the rectum may be even less pronounced. We therefore also performed an analysis after splitting our cohort in patients with tumors in the colon vs. the rectum. In this analysis, association between tumor *LDB1* expression and Wnt pathway gene expression remained significant for samples derived from the colon, but not for samples derived from the rectum (Table 3B), again stressing the increased effects of *LDB1* in the proximal colon as opposed to the distal colorectum.

**Table 3:** Correlation between tumor LDB1 expression and Wnt signaling-associated genes expression in CRC patients

A. Location	Parameter	*CTNNB1*	*AXIN2*	*MYC*	*CCND1*
**All samples**	Pearson correlation	0.471	0.465	0.448	0.446
	*p* value	< 0.001	< 0.001	< 0.001	< 0.001
**Proximal**	Pearson correlation	0.493	0.766	0.709	0.538
	*p* value	0.147	0.010	0.022	0.109
**Distal**	Pearson correlation	0.447	0.485	0.410	0.460
	*p* value	0.001	< 0.001	0.003	0.001

B. Location	Parameter	*CTNNB1*	*AXIN2*	*MYC*	*CCND1*
**All samples**	Pearson correlation	0.471	0.465	0.448	0.446
	*p* value	< 0.001	< 0.001	< 0.001	< 0.001
**Colon**	Pearson correlation	0.636	0.739	0.659	0.766
	*p* value	0.001	< 0.001	< 0.001	< 0.001
**Rectum**	Pearson correlation	0.322	0.273	0.274	0.171
	*p* value	0.059	0.113	0.112	0.326

^A^Proximal and distal samples were analyzed separately.

^B^Colon and rectum samples were analyzed separately.

## DISCUSSION

CRC is among the most commonly investigated tumor entities [2, 17, 33, 34]. The most prominent signaling pathway in CRC is Wnt signaling, its activation (most frequently by inactivating APC mutations) usually initiates the adenoma-carcinoma sequence and thus colorectal tumorigenesis [35]. Previous data from mouse models demonstrated a prominent role of *Ldb1* in hepatocellular carcinoma and intestinal homeostasis, indicated an inhibition of Wnt signaling by *Ldb1* and hence a tumorsuppressive role of *Ldb1* overexpression [14, 15]. As the majority of CRCs are heavily dependent on Wnt signaling, we hypothesized that *LDB1* may have a role in human CRC as well. More precisely, we aimed to show the role of *LDB1* as a tumor suppressor in CRC.

Unsurprisingly, the expression of *LDB1* in our patients proved to be heterogeneous. Surprisingly, however, patients with lower *LDB1* expression in the tumor experienced improved overall and metastasis-free survival, thus demonstrating a negative prognostic effect of *LDB1* expression in human CRC. These results could be reproduced in two independent, large cohorts of patients (Affymetrix and TCGA samples), therefore strongly validating our results. In addition, qPCR expression profiling showed higher expression levels of *CTNNB1*, *AXIN2*, *MYC* and *CCND1* in patients with *LDB1* overexpression, suggesting an increased Wnt signaling activity in these tumors and supporting the clinical data reported above.

These findings are in contradiction to previous data [13–15], which indicated a suppressive effect of *Ldb1* on the Wnt signaling pathway. However, these data were from mouse models only; it is conceivable that murine *Ldb1* has different effects and targets than human *LDB1*. Another explanation of the contradictory results of many studies on *Ldb1* may be organspecific effects of *Ldb1*: While inactivation of *Ldb1* led to an increase in tumor growth in murine hepatocellular carcinoma [15], its overexpression in mammary glands promoted breast tumorigenesis [36, 37]. In addition, widespread *LDB1* expression has been demonstrated in embryonic epithelial tissues [38, 39] and at the invasive front of squamous cell carcinoma of the head and neck [40], preventing differentiation of *LDB1*-overexpressing cells and again suggesting tumor-promoting effects of *LDB1*. These findings strongly suggest organ-specific effects of *Ldb1* and further support our data identifying *LDB1* as a negative prognostic factor in CRC.

To further validate *LDB1* as a tumor-promoting factor in CRC, we initiated a series of *in vitro* experiments using lentiviral vectors to overexpress *LDB1* in human CRC cell lines. We observed differential effects; however, most results again pointed towards an activation of the Wnt signaling pathway in human CRC, supporting the results seen in patient samples. Interestingly, the 4 genes we used as indicators of Wnt signaling activity were not uniformly regulated upon *LDB1* overexpression, indicating incomplete activation of the Wnt signaling pathway, which has recently be described in other tumor entities and transcription factors as well [41].

There are currently no studies available investigating the correlation of *LDB1* overexpression with metastatic activity; however, *LDB1* overexpression at the invasive front of squamous cell carcinomas of the head and neck suggests a connection between *LDB1* overexpression and metastasis [40]. In the present study, patients with *LDB1* overexpression in the tumor tissue displayed worse overall survival and metastasis-free survival. These results indicate that CRC tumors with low expression of *LDB1* metastasized later than tumors with a higher expression, indicating pro-metastatic features of *LDB1* in CRC. This finding was also supported by the other two clinical cohorts and is well in line with above mentioned data from human SCC of the head and neck [40].

Colorectal cancer is usually classified as a single entity despite data showing distinct molecular, pathological and clinical features of proximal and distal colon cancers [28–30]. This differentiation is embryologically well justified as the colorectum develops from both the midgut (ascending and transverse colon) and the hindgut (descending and sigmoid colon and rectum). Above mentioned studies demonstrated increased activation of the Wnt pathway and *MYC* expression in distal colorectal carcinomas in comparison with proximal tumors. Given this differential role of Wnt signaling in CRC and the effects of *LDB1* overexpression on Wnt signaling, we hypothesized that the role of *LDB1* may be different in proximal and distal CRC. We therefore analyzed the clinical data of our patient cohort as well as previously published patients according to the location of the tumors and were able to demonstrate a larger effect of *LDB1* overexpression in proximal CRC compared to distal CRC. In addition, the association between *LDB1* expression and Wnt signaling activity was stronger in tumors of the colon than rectal tumors. These differential roles of *LDB1* according to tumor location may be explained by the varying levels of Wnt activity on proximal and distal CRC: Proximal carcinomas upregulate the Wnt pathway to a lesser extent than distal tumors; therefore, *LDB1* may upregulate Wnt in proximal tumors more easily than in distal tumors, which already exhibit high Wnt signaling activity. The relatively increased expression of Wnt pathway genes in the *LDB1-*overexpressing, proximal (colon) cancer samples analyzed here may be a result of the higher *LDB1* expression; this in turn can explain the increased clinical effects of *LDB1* overexpression in proximal CRC samples.

To study the influence of *LDB1* on malignant features such as proliferation, invasiveness and cell cycle dysregulation, HCT116, DLD1, SW480 and SW620 cells were infected with a third generation lentivirus to overexpress *LDB1*. Although the gene expression and phenotypic changes after the upregulation of *LDB1* did not show a unique pattern, it was possible to recognize that HCT116_*LDB1-High*_ and DLD1_*LDB1-High*_ cells developed a similar phenotype with increased proliferation and augmented the expression of *CCND1*. SW480_*LDB1-High*_ and SW620_*LDB1-High*_ cells constituted a second phenotype, exhibited an upregulation of Wnt pathway genes including a marked overexpression of *MYC*, reduced proliferation rate and increased invasivity.

Generally, the Wnt signaling activity on proximal colon tumors is lower compared to the Wnt signaling activity in distal colon tumors [31, 32]. The site of origin (proximal vs. distal colon) of the cell lines is unknown; however, based on the Wnt signaling activity they may correspond to proximal (group 1) and distal colon (group 2). The overexpression of *LDB1* resulted in an increase in proliferation and Wnt signaling activity in group 1, while it reduced the proliferation in group 2. This supports our clinical findings, indicating a greater role of *LDB1* overexpression in tumors of the proximal colon. On the other hand, *LDB1* overexpression also has an effect in distal CRC, albeit weaker. In addition to decreased proliferation, the effects of *LDB1* overexpression on the group 2 cell lines included increased invasiveness which may partly explain the clinical findings.

In conclusion, *LDB1* overexpression is a negative prognostic factor in colorectal cancer. Its effects are stronger in tumors of the proximal colon, which may be due to comparatively low intrinsic Wnt signaling activity in these tumors which is stimulated by *LDB1* overexpression. *In vitro*, *LDB1* overexpression has differential effects on human CRC cell lines, which is again dependent on their intrinsic Wnt activity. Limitations of this study include its retrospective nature. However, the clear prognostic effects of *LDB1* overexpression demonstrated in this work clearly indicate the need for further studies. Given the unclear role of adjuvant treatment in stage II CRC patients and the prognostic value of *LDB1* regarding the prediction of metastasis, a prospective study in stage II CRC may establish *LDB1* as a valuable biomarker in nonmetastatic CRC.

## MATERIALS AND METHODS

### Patient samples

Clinical data and samples are being collected continuously at the Department of Surgery of University Hospital Heidelberg; our management of patients with colorectal cancer has been published in detail before [16, 17]. Tumor and mucosa samples were collected from 59 CRC patients (of all UICC stages), which underwent tumor resection at the Department of General, Visceral and Transplantation Surgery at the University Hospital in Heidelberg between October 2006 and July 2012. The study was approved by the independent ethics committee of the University of Heidelberg. All patients provided written informed consent prior to surgery. This patient cohort is henceforth referred to as the Heidelberg cohort.

### Affymetrix CRC cohort

A database of publicly available colorectal cancer patient samples measured by Affymetrix gene chips was set up as described previously for gastric cancer [18]. In brief, gene chip datasets with transcriptome-wide gene expression data generated by Affymetrix gene arrays and available survival data were identified in the Gene Expression Omnibus repository (www.ncbi.nlm.nih.gov/geo/). Samples were MAS5 normalized. We selected the probe set 35160_at for *LDB1*. Survival analysis was performed by employing Cox proportional hazard regression in the R statistical environment (www.r-project.org) as described previously [19]. Kaplan–Meier survival plot, and the hazard ratio with 95% confidence intervals and log-rank *P* value were plotted using the library “survival”.

## TCGA CRC COHORT

Colorectal cancer patients measured by RNA-seq were published in The Cancer Genome Atlas (TCGA) [3]. Pre-processed level 3 data generated using Illumina HiSeq 2000 RNA Sequencing V2 was used. For each sample, the expression level was determined using a combination of MapSplice and RSEM. The individual sample files were merged in R using the plyr package [20]. Survival analysis was performed as for the Affymetrix cohort.

### Cell culture

CRC cell lines were obtained from ATCC (HCT116, DLD1, SW480, SW620) and cultured at 37ºC with 5% CO_2_ and 95% humidity. DMEM (Thermo Fisher, Waltham, USA) and RPMI (Thermo Fisher, Waltham, USA) media were supplemented with 10% fetal bovine serum (FBS) (Thermo Fisher, Waltham, USA) and no antibiotics were used. Cell lines were regularly screened for Mycoplasma contamination; the cell line authenticity was confirmed regularly.

### *LDB1* overexpression

Cell lines constitutively overexpressing *LDB1* were generated by transduction with a third generation lentiviral vector system [21]. For virus production, HEK293T cells were cultured in DMEM medium with 10 % heat inactivated FBS. A mixture of 3 mL OptiMEM^®^ (Thermo Fisher, Waltham, USA) medium, 28 μL Lipofectamin^®^ 2000 (Thermo Fisher, Waltham, USA), 4 μg pMDLg/pRRE (was a gift from Didier Trono, Addgene plasmid # 12251), 2 μg pRSVRev (Addgene plasmid # 12253), 2 μg pMD2.G (Addgene plasmid # 12259) and 6 μg EX-Z6214-Lv205 (GeneCopoeia, Rockville, USA) was added dropwise to transfect HEK293T cells. After 24 hours, medium was replaced with fresh medium and incubated for another 24 hours. The virus-enriched medium was collected, filtered and mixed with polybrene (8 μg/mL final concentration). 1 × 10^5^ cells from each CRC cell line were seeded per well on 6-well plates and incubated with the virus solution for seven hours. After 48 hours, transduced cells were selected with puromycin (final concentration 10 μg/mL). To select brightly GFP expressing cells, CRC cell lines were sorted with a FACS ARIA II (BD Biosciences, San Jose, USA). Sorted cells were grown in medium with 10% FBS and 1% penicillin/streptomycin. Control cell lines were generated similarly using a modified version of the EXZ6214Lv205 vector, lacking the *LDB1* gene.

### Plasmid amplification

One Shot^®^ TOP10 chemically competent *E. coli* cells (Thermo Fisher, Waltham, USA) were transformed following the manufacturer's instructions, adding 2 μL from the corresponding plasmids. Plasmid DNA was isolated with the Qiagen EndoFree^®^ Maxi Kit (Qiagen, Hilden, Germany), following the manufacturer's protocol. DNA was eluted with nuclease-free water and concentration was adjusted to reach 1 μg/μL.

### RNA isolation

Tissue samples were fragmented using the TissueLyser II (Qiagen, Hilden, Germany). The NucleoSpin^®^ RNA Kit (Macherey-Nagel, Düren, Germany) was used for RNA isolation following the manufacturer's instructions. Tissue fragments were inserted into microcentrifuge tubes containing 700 μL buffer RA1 and one 5 mm stainless steel bead (Qiagen, Hilden, Germany). Samples were shaken twice at 30 Hz for 2 minutes. Cell lines pellets were lysed with 350 μL buffer RA1. RNA was eluted with 40 μL RNAase-free water and concentration was measured using a NanoVue™ Plus Spectrophotometer (GE Healthcare, Piscataway, USA).

### cDNA synthesis

The High-Capacity cDNA Reverse Transcription Kit (Thermo Fisher, Waltham, USA) was used for cDNA synthesis. For each reaction, 1 μg of RNA was mixed with 2 μL RT Buffer, 2 μL RT random primers, 0.8 μL dNTP Mix, 1 μL MultiScribe™ Reverse Transcriptase and RNAase-free water to complete 20 μL. Tubes were incubated in a thermocycler at 25ºC for 10 minutes, at 37ºC for 120 minutes and at 85ºC for 5 minutes. Tissue samples were diluted with Nuclease-free water in a 1:5 ratio and cell lines in a 1:10 ratio.

### Quantitative PCRs

Methods have been described before [22, 23]. Briefly, quantitative PCRs were performed using the StepOnePlus™ RealTime PCR System (Thermo Fisher, Waltham, USA). One reaction consisted in 2.5 μL cDNA, 5 μL Power SYBR^®^ Green PCR Master Mix (Thermo Fisher, Waltham, USA), 0.5 μL from each 5 μM primer (forward and reverse) and 1.5 μL RNAase-free water. The following primers were designed using Primer3Plus (5′ to 3′): *GAPDH*_fwd GACCCCTTCATTGACCTCAAC; *GAPDH*_rev TTGATTTTGGAGGGATCTCG; *LDB1*_fwd TGTATCCGCCTACATACCTG; *LDB1*_rev TGGTCAA CATGGCATCATCC; *CTNNB1*_fwd TTCCGAATGT CTGAGGACAAG; *CTNNB1*_rev TGGGCACCAA TATCAAGTCC; *AXIN2*_fwd TGGCTATGTCTTT GCACCAG; *AXIN2*_rev TGTTTCTTACTGCCCACACG; *MYC*_fwd TGCTCCATGAGGAGACACC; *MYC*_rev GATCCAGACTCTGACCTTTTGC; *CCND1*_fwd TCCT CTCCAGAGTGATCAAGTG; *CCND1*_rev TTGGGG TCCATGTTCTGC. Holding stage was set at 50ºC for 2 minutes and at 95ºC for 10 minutes. Cycling stage at 95ºC for 30 seconds and at 60ºC for 1 minute repeated 40 times. Melt curve stage was set at 95ºC for 15 seconds and ramp with an 0.2ºC increase from 65ºC to 97ºC. Cycle thresholds (Ct) values were calculated using the AB StepOne™ v2.1 software (Thermo Fisher, Waltham, USA). Tissue Ct values were first normalized to *GAPDH* and then to mucosa using the ΔΔCt equation:

ΔΔCt=(Ct GAPDH Tumor − CT target gene Tumor)−(Ct GAPDH Mucosa − CT target gene Mucosa)

Ct values from cell lines were normalized to *GAPDH*. The fold change of these genes was calculated using the 2^ΔCt^ equation.

$$2^{\Delta Ct}=2^{\left(Ct\;GAPDH\;-\;CT\;target\;gene\right)}$$

Genetic modified CRC cell lines were first normalized to *GAPDH* and then normalized with the wildtype cell lines, using the following equation:

$$2^{\Delta \Delta Ct}=2^{\begin{array}{l} \left(Ct\;GAPDH\;modified\;cells\;-\;Ct\;target\;modified\;cells\right)-\\ \left(Ct\;GAPDH\;wildtype\;cells\;-\;Ct\;target\;wildtype\;cells\right) \end{array}}$$

### Cell proliferation

Cell lines were seeded onto 24-well plates and incubated for three days at 37ºC with 5% CO_2_ and 95% humidity. Cells were then trypsinized and resuspended in 1 mL of medium. Ten microliters from the cell suspension were pipetted per pocket into counting slides. Cells were counted with an automatic cell-counter.

### TOP/FOP functional Wnt activation assay

Ten to twenty thousand cells were seeded per well onto 96-well plates. After 24 hours' incubation, cells were transfected (per well) with 19.6 μL OptiMEM^®^ (Thermo Fisher, Waltham, USA), 0.2 μL Lipofectamin^®^2000 (Thermo Fisher, Waltham, USA), 10 ηg Renilla plasmid [24] and 100 ηg TOP or FOP plasmids [25, 26]. Plates were incubated overnight at 37°C. Luminescence produced by Firefly and Renilla luciferase was measured with a Tecan GENios spectrophotometer (Tecan, Männedorf, Switzerland) using the DualGlo^®^ Luciferase Assay System (Promega, Madison, USA). Firefly was first normalized to Renilla and then the ratio between TOP and FOP was calculated.

### Cell cycle analysis

General flow cytometry methods of our group have been published in detail before [27]. Briefly, cells were seeded onto 24-well plates. After three days in culture, supernatant and cells were collected into flow cytometry tubes. Pellets were resuspended with 1 mL ice-cold 70% ethanol and incubated at 4°C overnight. Samples were centrifuged and cells were washed twice with 1 mL PBS. To digest RNA, each pellet was incubated with 100 μL of 0.2 mg/mL RNase A solution (Sigma-Aldrich, St Louis, USA) at 37°C for 1 hour. Cells were incubated with 10 μL 1 mg/mL Propidium Iodide (Sigma-Aldrich, St Louis, USA). Flow cytometry was performed with the BD FACSCalibur™ (BD Biosciences, San Jose, USA). In total 10,000 events were counted per cell sample. Cell counts were analyzed with the WinMDI 2.9 software (The Scripps Research Institute, La Jolla, USA) to determine the percentage of cells per cell cycle phase.

### Scratch assay

One to two million cells were seeded per well onto 6-well plates. After 24 hours, scratches were made on each well with a 100 μl pipet tip. Perpendicular lines were marked bellow the bottom of the plate to determinate a specific place. Pictures were made after 24 hours with a Leica DMI3000B microscope (Leica Microsystems, Wetzlar, Germany) using the Leica Application Suite v3.6 software (Leica Microsystems, Wetzlar, Germany). The distance between the two edges was measured and shortening was calculated using the same software.

### Statistical analysis

Survival/recurrence rates were depicted using Kaplan-Meier curves and compared using the log-rank test. Results from *in vitro* experiments were compared using the Student's independent samples *t*-test (to compare means) and bivariate correlation (Pearson correlation). *P* values < 0.05 were considered statistically significant. The following code was used in this article to display significance levels: (*) *p* < 0.05, (**) *p* < 0.01, (***) *p* < 0.001.

## SUPPLEMENTARY MATERIALS FIGURES AND TABLES



## References

[1] Siegel RL, Miller KD, Jemal A (2016). Cancer statistics, 2016. CA Cancer J Clin.

[2] Weitz J, Koch M, Debus J, Hohler T, Galle PR, Buchler MW (2005). Colorectal cancer. Lancet.

[3] Cancer Genome Atlas N (2012). Comprehensive molecular characterization of human colon and rectal cancer. Nature.

[4] Bernatik O, Ganji RS, Dijksterhuis JP, Konik P, Cervenka I, Polonio T, Krejci P, Schulte G, Bryja V (2011). Sequential activation and inactivation of Dishevelled in the Wnt/beta-catenin pathway by casein kinases. J Biol Chem.

[5] Li VS, Ng SS, Boersema PJ, Low TY, Karthaus WR, Gerlach JP, Mohammed S, Heck AJ, Maurice MM, Mahmoudi T, Clevers H (2012). Wnt signaling through inhibition of beta-catenin degradation in an intact Axin1 complex. Cell.

[6] He TC, Sparks AB, Rago C, Hermeking H, Zawel L, da Costa LT, Morin PJ, Vogelstein B, Kinzler KW (1998). Identification of c-MYC as a target of the APC pathway. Science.

[7] Wielenga VJM, Smits R, Korinek V, Smit L, Kielman M, Fodde R, Clevers H, Pals ST (1999). Expression of CD44 in Apc and TcfMutant Mice Implies Regulation by the WNT Pathway. The American Journal of Pathology.

[8] Shtutman M, Zhurinsky J, Simcha I, Albanese C, D'Amico M, Pestell R, Ben-Ze'ev A (1999). The cyclin D1 gene is a target of the beta-catenin/LEF-1 pathway. P Natl Acad Sci USA.

[9] Lustig B, Jerchow B, Sachs M, Weiler S, Pietsch T, Karsten U, van de Wetering M, Clevers H, Schlag PM, Birchmeier W, Behrens J (2002). Negative Feedback Loop of Wnt Signaling through Upregulation of Conductin/Axin2 in Colorectal and Liver Tumors. Molecular and Cellular Biology.

[10] Matthews JM, Visvader JE (2003). LIM-domain-binding protein 1: a multifunctional cofactor that interacts with diverse proteins. EMBO Rep.

[11] Popperl H, Schmidt C, Wilson V, Hume CR, Dodd J, Krumlauf R, Beddington RS (1997). Misexpression of Cwnt8C in the mouse induces an ectopic embryonic axis and causes a truncation of the anterior neuroectoderm. Development.

[12] Borello U, Coletta M, Tajbakhsh S, Leyns L, De Robertis EM, Buckingham M, Cossu G (1999). Transplacental delivery of the Wnt antagonist Frzb1 inhibits development of caudal paraxial mesoderm and skeletal myogenesis in mouse embryos. Development.

[13] Mukhopadhyay M, Teufel A, Yamashita T, Agulnick AD, Chen L, Downs KM, Schindler A, Grinberg A, Huang SP, Dorward D, Westphal H (2003). Functional ablation of the mouse Ldb1 gene results in severe patterning defects during gastrulation. Development.

[14] Dey-Guha I, Mukhopadhyay M, Phillips M, Westphal H (2009). Role of ldb1 in adult intestinal homeostasis. Int J Biol Sci.

[15] Teufel A, Maass T, Strand S, Kanzler S, Galante T, Becker K, Strand D, Biesterfeld S, Westphal H, Galle PR (2010). Liver-specific Ldb1 deletion results in enhanced liver cancer development. J Hepatol.

[16] Bork U, Rahbari NN, Schölch S, Reissfelder C, Kahlert C, Buchler MW, Weitz J, Koch M (2015). Circulating tumour cells and outcome in non-metastatic colorectal cancer: a prospective study. Br J Cancer.

[17] Rahbari NN, Bork U, Schölch S, Reissfelder C, Thorlund K, Betzler A, Kahlert C, Schneider M, Ulrich AB, Buchler MW, Weitz J, Koch M (2016). Metastatic Spread Emerging From Liver Metastases of Colorectal Cancer: Does the Seed Leave the Soil Again?. Ann Surg.

[18] Szasz AM, Lanczky A, Nagy A, Forster S, Hark K, Green JE, Boussioutas A, Busuttil R, Szabo A, Gyorffy B (2016). Cross-validation of survival associated biomarkers in gastric cancer using transcriptomic data of 1,065 patients. Oncotarget.

[19] Mihaly Z, Kormos M, Lanczky A, Dank M, Budczies J, Szasz MA, Gyorffy B (2013). A meta-analysis of gene expression-based biomarkers predicting outcome after tamoxifen treatment in breast cancer. Breast Cancer Res Treat.

[20] Wickham H (2011). The Split-Apply-Combine Strategy for Data Analysis. Journal of Statistical Software.

[21] Salmon P, Trono D., Science E Design and Production of Human Immunodeficiency Virus-Derived Vectors. Cell Biology.

[22] Steinert G, Schölch S, Niemietz T, Iwata N, Garcia SA, Behrens B, Voigt A, Kloor M, Benner A, Bork U, Rahbari NN, Buchler MW, Stoecklein NH (2014). Immune escape and survival mechanisms in circulating tumor cells of colorectal cancer. Cancer research.

[23] Schölch S, Garcia SA, Iwata N, Niemietz T, Betzler AM, Nanduri LK, Bork U, Kahlert C, Thepkaysone ML, Swiersy A, Buchler MW, Reissfelder C, Weitz J (2016). Circulating tumor cells exhibit stem cell characteristics in an orthotopic mouse model of colorectal cancer. Oncotarget.

[24] Barker N, Clevers H (2006). Mining the Wnt pathway for cancer therapeutics. Nat Rev Drug Discov.

[25] van de Wetering M, Oosterwegel M, Dooijes D, Clevers H (1991). Identification and cloning of TCF-1, a T lymphocyte-specific transcription factor containing a sequence-specific HMG box. EMBO J.

[26] van de Wetering M, Cavallo R, Dooijes D, van Beest M, van Es J, Loureiro J, Ypma A, Hursh D, Jones T, Bejsovec A, Peifer M, Mortin M, Clevers H (1997). Armadillo coactivates transcription driven by the product of the Drosophila segment polarity gene dTCF. Cell.

[27] Schölch S, Rauber C, Tietz A, Rahbari NN, Bork U, Schmidt T, Kahlert C, Haberkorn U, Tomai MA, Lipson KE, Carretero R, Weitz J, Koch M (2015). Radiotherapy combined with TLR7/8 activation induces strong immune responses against gastrointestinal tumors. Oncotarget.

[28] Minoo P, Zlobec I, Peterson M, Terracciano L, Lugli A (2010). Characterization of rectal, proximal and distal colon cancers based on clinicopathological, molecular and protein profiles. Int J Oncol.

[29] Benedix F, Kube R, Meyer F, Schmidt U, Gastinger I, Lippert H, Colon/Rectum Carcinomas Study G (2010). Comparison of 17,641 patients with right- and left-sided colon cancer: differences in epidemiology, perioperative course, histology, and survival. Dis Colon Rectum.

[30] Missiaglia E, Jacobs B, D’Ario G, Di Narzo AF, Soneson C, Budinska E, Popovici V, Vecchione L, Gerster S, Yan P, Roth AD, Klingbiel D, Bosman FT (2014). Distal and proximal colon cancers differ in terms of molecular, pathological, and clinical features. Ann Oncol.

[31] Christie M, Jorissen RN, Mouradov D, Sakthianandeswaren A, Li S, Day F, Tsui C, Lipton L, Desai J, Jones IT, McLaughlin S, Ward RL, Hawkins NJ (2013). Different APC genotypes in proximal and distal sporadic colorectal cancers suggest distinct WNT/beta-catenin signalling thresholds for tumourigenesis. Oncogene.

[32] Albuquerque C, Baltazar C, Filipe B, Penha F, Pereira T, Smits R, Cravo M, Lage P, Fidalgo P, Claro I, Rodrigues P, Veiga I, Ramos JS (2010). Colorectal cancers show distinct mutation spectra in members of the canonical WNT signaling pathway according to their anatomical location and type of genetic instability. Genes Chromosomes Cancer.

[33] Schölch S, Bork U, Rahbari NN, Garcia S, Swiersy A, Betzler AM, Weitz J, Koch M (2014). Circulating tumor cells of colorectal cancer. Cancer Cell & Microenvironment.

[34] Steinert G, Schölch S, Koch M, Weitz J (2012). Biology and significance of circulating and disseminated tumour cells in colorectal cancer. Langenbecks Arch Surg.

[35] Saif MW, Chu E (2010). Biology of colorectal cancer. Cancer J.

[36] Salmans ML, Yu Z, Watanabe K, Cam E, Sun P, Smyth P, Dai X, Andersen B (2014). The co-factor of LIM domains (CLIM/LDB/NLI) maintains basal mammary epithelial stem cells and promotes breast tumorigenesis. PLoS genetics.

[37] Visvader JE, Venter D, Hahm K, Santamaria M, Sum EY, O’Reilly L, White D, Williams R, Armes J, Lindeman GJ (2001). The LIM domain gene LMO4 inhibits differentiation of mammary epithelial cells *in vitro* and is overexpressed in breast cancer. Proc Natl Acad Sci USA.

[38] Sugihara TM, Bach I, Kioussi C, Rosenfeld MG, Andersen B (1998). Mouse deformed epidermal autoregulatory factor 1 recruits a LIM domain factor, LMO-4, and CLIM coregulators. Proc Natl Acad Sci USA.

[39] Bach I, Carriere C, Ostendorff HP, Andersen B, Rosenfeld MG (1997). A family of LIM domain-associated cofactors confer transcriptional synergism between LIM and Otx homeodomain proteins. Genes Dev.

[40] Mizunuma H, Miyazawa J, Sanada K, Imai K (2003). The LIM-only protein, LMO4, and the LIM domain-binding protein, LDB1, expression in squamous cell carcinomas of the oral cavity. Br J Cancer.

[41] Chen W, Liang J, Huang L, Cai J, Lei Y, Lai J, Liang L, Zhang K (2016). Characterizing the activation of the Wnt signaling pathway in hilar cholangiocarcinoma using a tissue microarray approach. Eur J Histochem.

