# Supplementary material for: LDB1 overexpression is a negative prognostic factor in colorectal cancer
Source: Oncotarget. 2016 Oct 5;7(51):84258–70. doi: 10.18632/oncotarget.12481 (PMC5356660; doi:10.18632/oncotarget.12481)
Supplement: Supplementary file 1 [file oncotarget-07-84258-s001.pdf]

LDB1 overexpression is a negative prognostic factor in colorectal cancer

Supplementary Materials

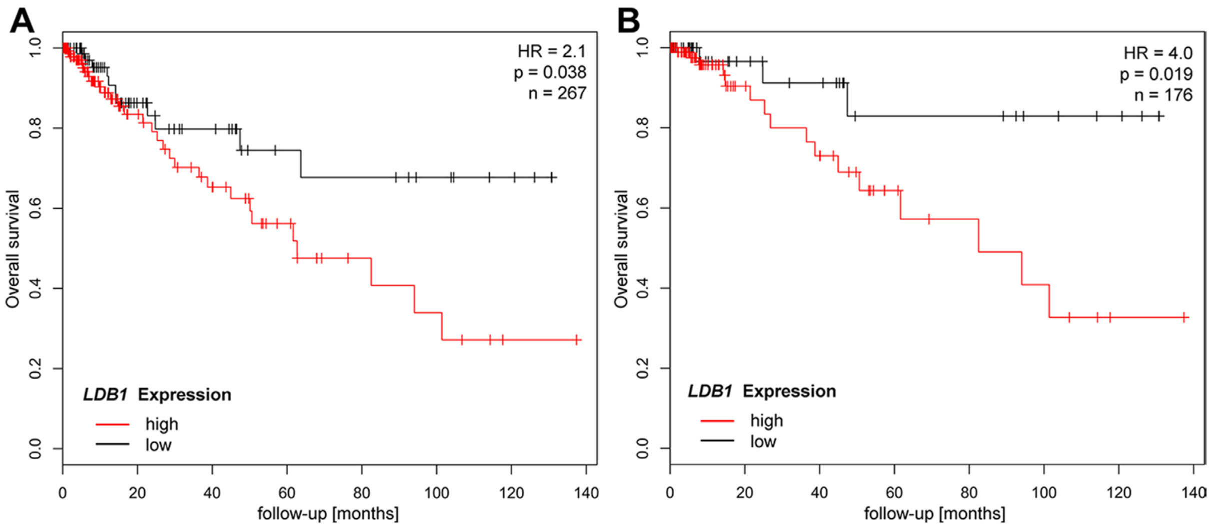

Supplementary Figure S1: Overall survival of patients in the TCGA cohort. *LDB1* overexpression leads to reduced overall survival in CRC patients of all stages (**A**, HR = 2.1, $p = 0.038$) and, much more pronounced, non-metastatic (M0) patients (**B**, HR = 4.0, $p = 0.019$).
